# Tikhon Efimovich Boldyrev (1900–1984): A Soviet epidemiologist's contributions to public health in 1950s China

**DOI:** 10.1177/09677720251398961

**Published:** 2025-11-21

**Authors:** Shanshan Gao

**Affiliations:** 1 Department of History and Philosophy of Science, 2152 University of Cambridge, Cambridge, UK

**Keywords:** epidemic prevention, health policies, soviet experts, Tikhon Efimovich Boldyrev, traditional Chinese medicine

## Abstract

Tikhon Efimovich Boldyrev was a prominent Soviet epidemiologist. During the Sino-Soviet collaboration of the 1950s, he played a key advisory role in shaping health policies in the newly established People's Republic of China. This article explores Boldyrev's life and his journey to China, where he served as Group Leader of the Soviet Experts and Chief Expert at the Chinese Ministry of Health from 1954 to 1956. During his tenure, Boldyrev authored twenty reports and proposals that influenced China's public health policy. His notable contributions included introducing and adapting the Soviet healthcare model to Chinese conditions; endorsing traditional Chinese medicine and advocating for its integration with modern medical science; and providing critical expertise in epidemic prevention, particularly in combating diseases such as schistosomiasis and plague. Drawing on Boldyrev's work completed in China, along with Chinese-language government reports, press coverage, and professional journals, this article brings renewed attention to his important yet often overlooked contributions to public health in 1950s China.

## Introduction

**Figure 1.**
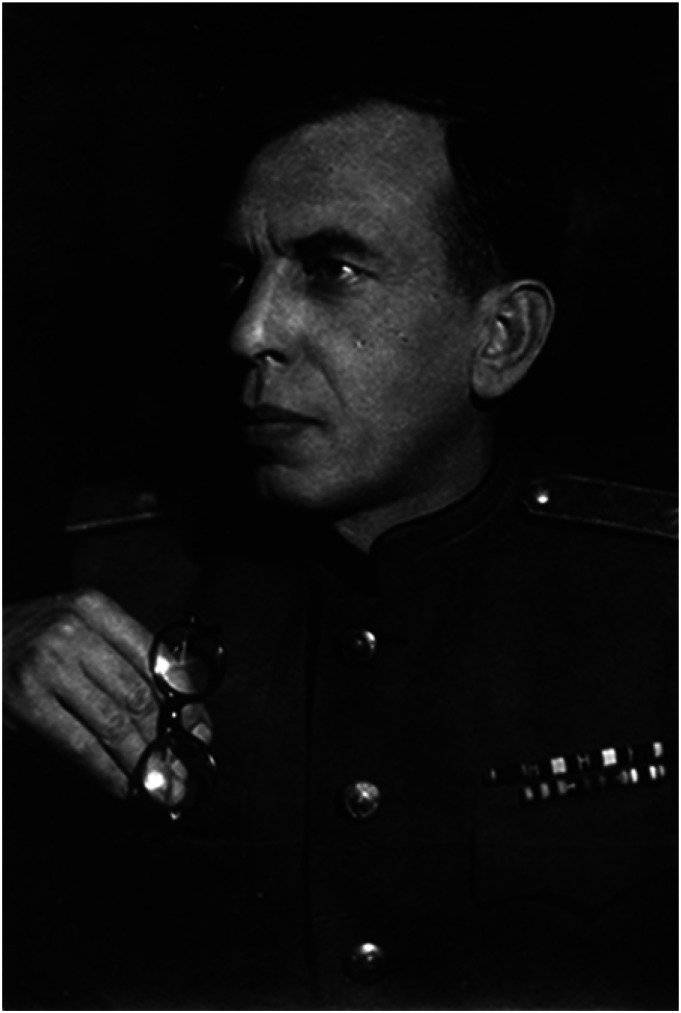
T. E. Boldyrev (1900–1984).

Tikhon Efimovich Boldyrev (Figure 1) was a renowned epidemiologist in the Union of Soviet Socialist Republics (USSR, 1922–1991). His career began when he enlisted as a soldier in the Red Army in 1919. Upon completing his studies at the Military Medical Academy in Saint Petersburg in 1926, he served as a brigade surgeon, steadily rising to the rank of Major-General of Medical Services by 1943.^1^ Boldyrev dedicated his life to medical research, focusing primarily on epidemiology and military medicine. He authored and edited over 15 books on these two subjects and held several influential academic and leadership positions. During World War II (1939–1945), he led the Epidemiology Department of the Kuibyshev Military Medical Academy from 1939 to 1941 before serving as the Head of the Anti-Epidemic Directorate within the Main Medical Directorate from 1941 to 1945. From 1945 to 1947, he was appointed as the Chief Epidemiologist for the Army, and from 1947 to 1953, he took on the responsibilities of Deputy Minister of Public Health and Chief Medical Inspector of the USSR. After suffering a myocardial infarction and declining health, Boldyrev left the front line and transitioned to the Reserve Army in 1953. From 1954 to 1956, he held the position of Group Leader of the Soviet Experts and Chief Expert at the Chinese Ministry of Health in Beijing, where he contributed to public health initiatives in China.^2^ After returning from China, Boldyrev headed the Epidemiology Departments at two medical institutes in the USSR, positions he held until his retirement in 1961.^3^

Scholarly articles by Russian researchers, based exclusively on Russian sources, highlight Boldyrev's leadership in sanitary and epidemiological services during World War II, drawing a clear connection between his wartime experiences and his subsequent impact on the USSR's public health, showcasing how his military service influenced his professional paths.^4^ Although widely recognised in Russia as a distinguished healthcare organiser and specialist, Boldyrev's contributory role in the development of health policies in the newly established People's Republic of China (PRC) during the 1950s has been largely overlooked. A thorough evaluation of Boldyrev's contributions in China has been hindered by language barriers, as relevant records exist primarily in Chinese, preventing many historians from providing a comprehensive analysis of his notable works. Additionally, Boldyrev's life, achievements, and influence are primarily documented in Russian, which leaves them relatively obscure to both Chinese and English-language scholarship. To address this research gap, this present article aims to illuminate both Boldyrev's influential role in Sino-Soviet public health cooperation and his contributions to shaping health policies in the PRC.

## Boldyrev's journey to China

In August 1945, at the close of World War II, the USSR's Red Army advanced into Northeast China, then the Japanese puppet state of Manchukuo (1932–1945), a region prone to plague outbreaks. Subsequently, control of much of Northeast China was transferred from the USSR to the Chinese Communist Party (CCP). On September 8, 1948, Lin Biao (1907–1971), Commander of CCP's Northeast Military Region, corresponded directly with Soviet leader Joseph Stalin (1878–1953), requesting the deployment of a significant number of economic advisors and experts to assist in the post-war recovery of Northeast China.^5^ In June 1949, a delegation from CCP's Central Committee visited the USSR. On their return to China, they brought with them 220 senior Soviet economic officials and engineers.^6^

From 1947 to 1949, the USSR, noted for its early development of the theory of natural plague foci and experience in plague prevention and control, annually deployed epidemic prevention expert teams to CCP-controlled Northeast China to address plague outbreaks.^7^ In August 1949, a group of Soviet medical experts arrived at Shenyang's China Medical University, launching the ‘Learning from the Soviet Union’ initiative in Northeast China.^8^ Following the establishment of the PRC in October 1949, a plague outbreak occurred in Northern Manchuria, near Beijing, the new capital of Soviet China. Mao Zedong (1893–1976), chairman of the CCP, urgently appealed to Stalin for assistance. Stalin arranged for the immediate airlifting of vaccines, serum, and an epidemic prevention expert team to Beijing to address the crisis. The outbreak was successfully contained by November 1949.^9^ At a conference celebrating the triumph over the Northern Manchurian plague, Chinese citizens delivered the carefully orchestrated cheer, ‘Long live the Sino-Soviet friendship, long live Stalin!’^10^ This episode marked the symbolic beginning of Sino-Soviet medical cooperation.

In the early years of the PRC, the close Sino-Soviet relationship played a pivotal role in shaping the nation's development. Lacking experience in governing a modern socialist state, the newly founded PRC turned to the USSR for guidance, adopting its Stalinist model and relying heavily on Soviet expertise. The outbreak of the Korean War (1950–1953) further deepened China's reliance on the USSR, sparking a nationwide trend of ‘Sovietisation’. Throughout the 1950s, an estimated 18,000 Soviet experts worked in China, providing vital support in fields such as heavy industry, agriculture, education, and medicine. It was within this broader context that Boldyrev carried out his work in China. The assistance lasted until 1960, when the Sino-Soviet split prompted a sudden withdrawal of all Soviet experts, bringing this era of collaboration to an abrupt end.^11^

Boldyrev and other Soviet experts were regarded as esteemed guests during their stay in China. During his two-year tenure (1954–1956) as the third-term Chief Expert in the Ministry of Health and leader of the Soviet expert group, Boldyrev earned a reputation as a highly competent specialist.^12^ In recognition of his contributions to the PRC, he was awarded the prestigious ‘Sino-Soviet Friendship’ medal and a certificate of appreciation from the Chinese Government.^13^ While in China, Boldyrev formed many close friendships with his Chinese colleagues. Today, the Tambov Museum of the History of Medicine in Russia preserves a collection of correspondence between Boldyrev and his Chinese acquaintances. Written in Chinese and accompanied by Russian translations, these letters are adorned with intricate drawings and appliqués that feature traditional Chinese motifs, such as dragons and scenic regional landscapes.^1^

**Figure 2.**
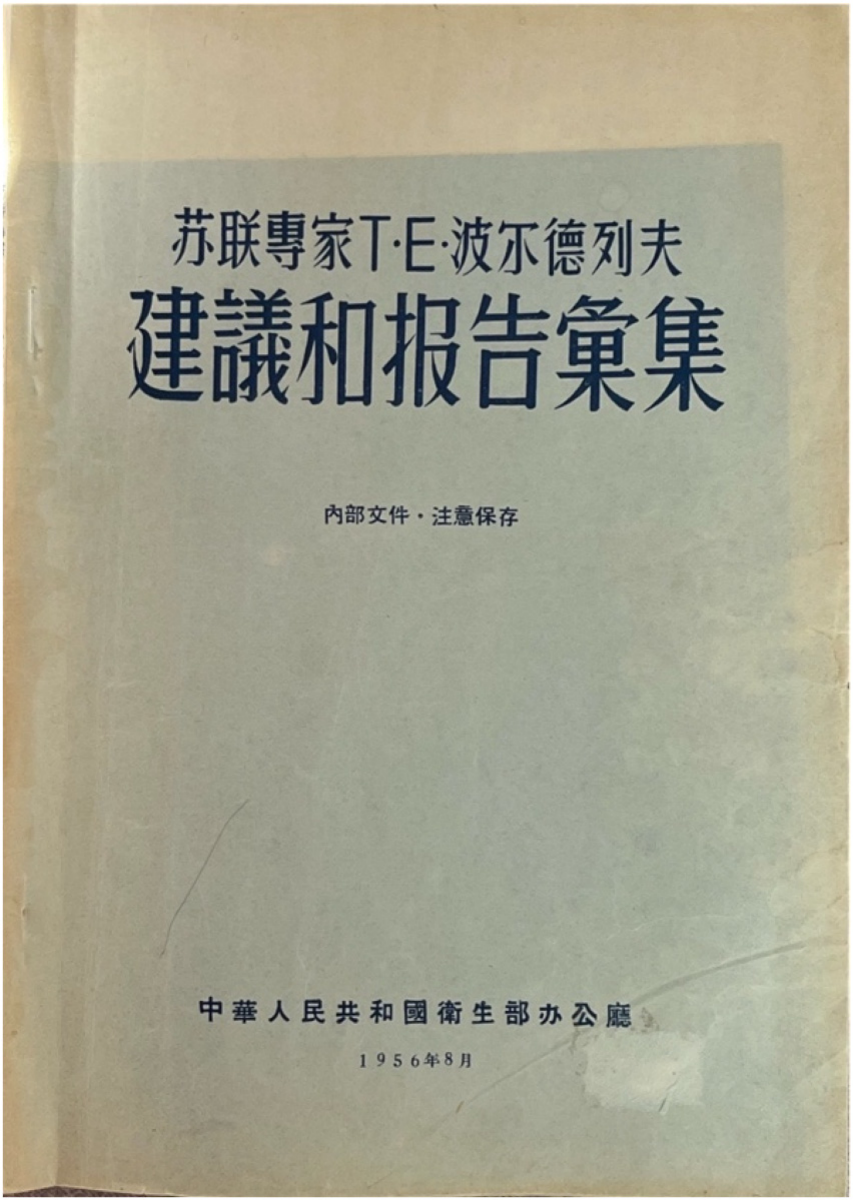
*The Collected Suggestions and Reports by the Soviet Expert T.E. Boldyrev*, 1956. With the warning ‘Internal Document, Preserve Carefully’ printed on the book cover.

Between April 1954 and January 1956, following field research across multiple sites, Boldyrev submitted nine proposals and eleven reports to the Chinese central government, addressing a wide range of healthcare-related issues. His recommendations included transplanting the Soviet model of preventive health supervision into China, introducing the USSR's higher medical education framework along with suggestions for improving China's scientific research, and implementing a Soviet-style worker-centric national healthcare system. Boldyrev also formulated foundational principles and regulations for the Chinese Ministry of Health, offered guidance on disease control strategies and sanitation standards in both rural and urban areas, as well as developed plans for the prevention and management of diseases such as schistosomiasis, plague, malaria, and encephalitis. Originally written in Russian, these reports were translated into Chinese and compiled into a book titled *The Collected Suggestions and Reports by the Soviet Expert T.E. Boldyrev*, officially published by the Ministry of Health of the PRC in August 1956 (Figure 2).^14^ This publication was classified as an ‘internal document’, restricting its distribution to select health officials for reference purposes rather than public circulation. In addition to this book, Boldyrev's insights were also published in *People's Daily*, the *National Medical Journal of China*, and included in two further essay collections.^15^ His contributions to China's health policy development were particularly impactful in three key areas: (1) bringing the Soviet healthcare model to China, (2) advocating for the use of traditional Chinese medicine, and (3) providing expertise on epidemic prevention. The following sections will examine each of these three pivotal contributions in detail.

## Introducing the Soviet healthcare model to China

From the founding of the PRC in October 1949 until the end of 1953, the health and medical policies previously employed by the CCP in its army and controlled regions were largely maintained. In 1953, following the Korean War armistice, the preliminary completion of economic recovery, and the political purge of the ‘King of Northeast China’ Gao Gang (1905–1954) and his so-called ‘Northeast Party’ members, particularly within the Ministry of Health, Mao turned his focus to the medical field. That same year, Mao declared: ‘China has to learn the most advanced science and technology from the USSR’,^16^ a directive that extended to the field of medicine. Against this backdrop, Boldyrev arrived in China in 1954 at just the right time. His expertise was highly valued by the Ministry of Health. Between April 1954 and May 1955, he delivered eight speeches introducing the USSR's medical system to the Chinese at conferences organised by the Ministry of Health.

Boldyrev's speeches provided insights into the Soviet healthcare system and how it could be potentially adapted for China. The topics his speeches covered can be divided into two main areas. The first addressed the historical development and contemporary status of the USSR's medical system, including medical education, scientific research, healthcare services for workers, urban construction and hygiene standards, as well as preventive hygiene supervision, with a particular emphasis on changes from the 1910s to the 1940s. The second focused on formulating regulations and general principles for the Ministry of Health and rural healthcare services in China, addressing issues within China's medical system, and exploring ways to adapt Soviet practices to Chinese conditions.

In his speeches, Boldyrev emphasised the importance of incorporating ideological and political education into the training of medical professionals. He highlighted the necessity for medical personnel to acquire a solid understanding of materialist philosophy, particularly dialectical materialism, drawing on key texts such as Joseph Stalin's *Dialectical and Historical Materialism*, as taught in the Soviet education system, and Mao's *On Practice* and *On Contradiction*. He underscored the need for doctors in all specialities to receive collective ideological education conducted by medical school principals, schools’ Party leaders, and other social organisations. Following Boldyrev's recommendations, political indoctrination was institutionalised as a foundational component of Chinese medical education.

To address the urgent need for more medical professionals in the newly established PRC, Boldyrev suggested shortening training programs. He referenced the USSR's experience during World War I (1914–1918) and the Russian Civil War (1918–1922), when senior medical school students were mobilised to join the workforce. Although these students had not yet obtained doctor certifications, they still served in roles equivalent to doctors. Drawing from these precedents, he proposed that medical assistants could be trained within ten months, while nurse training could be completed within three to five months. Furthermore, he suggested that individuals without prior medical education could gain hands-on clinical experience by working as hospital nurses. These recommendations significantly accelerated China's medical training pipeline. Starting in 1954, most Chinese medical continuation schools reduced their programme durations from one year to six months.^17^

China's First Five-Year Plan, launched in 1953 with a focus on prioritising the development of heavy industry, was deeply influenced by the Soviet model and its experts. As Boldyrev stated in May 1954:

Building a strong heavy industry sector was designated as the nation's top priority. Successfully accomplishing this [First Five-Year Plan] task would promote the growth of other sectors of the national economy and enhance national defence. The continued development of the USSR's national economy vividly demonstrated the validity of prioritising heavy industry. […] While fulfilling this task, healthcare institutions were tasked to prioritise providing medical and sanitary services to workers.^18^

Boldyrev praised China's ability to mobilise large numbers of people for health campaigns. He cited the patriotic health campaigns he observed in mines and factories across Northeast China as examples of efforts that significantly contributed to protecting workers’ health. He also encouraged China to draw from the USSR's experience in training large numbers of workers in industrial enterprises to perform self-help and mutual aid tasks. However, he cautioned:

This does not imply training workers to become medical practitioners. Anyone who assumes this is about creating barefoot doctors among workers is mistaken. Human life is invaluable, and medical treatment cannot be entrusted to individuals with only minimal medical knowledge.^19^

Ironically, although Boldyrev cautioned against rapid changes and proposed detailed strategies for the gradual improvement of rural healthcare in China, his suggestions indirectly contributed to the emergence of the nationwide Barefoot Doctors Movement (1968–1983) during the Cultural Revolution (1966–1976). This movement trained farmers to serve as primary healthcare providers in rural areas, fundamentally transformed village-level medicine and reshaped the medical landscape of post-socialist China.^20^

## Endorse traditional Chinese medicine

During the preceding Late Qing and Republican periods (1891–1949), a movement advocating for the abolition of Chinese medicine emerged in society, fueled by increasing scepticism toward Chinese medical practice and a growing preference for modern Western biomedicine. In contrast, under Mao's leadership, the CCP, along with its army and base areas, initiated an alternative movement that promoted the integration of Chinese and Western medicine.^21^ After the PRC's founding in 1949, this integrative approach was institutionalised and carried forward, solidifying the coexistence of Chinese and Western medicine and shaping a dualistic healthcare system. In September 1954, Boldyrev was invited by Xu Yunbei (1914–2018), Vice Minister of Health, to share his views on traditional Chinese medicine.

Boldyrev stated that he regarded traditional Chinese medicine as the oldest medical system in the world and an invaluable component of China's cultural heritage, deserving of preservation and continuous study. He criticised biomedical doctors for their dismissive attitude toward Chinese medicine, labelling their disdain ‘a serious ideological error’. Boldyrev argued that such disregard for the collective wisdom of the people reflected bourgeois thinking and cosmopolitanism—a mindset that assumed Chinese people were incapable of genuine innovation or creation and which believed that only foreigners were capable of such achievements. He went so far as to compare those who opposed Chinese medicine to individuals who ‘have forgotten their roots and disowned their parents’, a moral flourish that left little room for defence.

After decades of war, China entered the early 1950s with a severe shortage of biomedical doctors in both urban and rural areas. Of the 341,753 medical personnel working nationwide at that time, 306,583 (89.7%) were in rural areas. The majority of those skilled and experienced rural healthcare providers were practitioners of Chinese medicine. Knowing this reality, Boldyrev argued that China should fully utilise its available Chinese medicine practitioners to enhance its healthcare services.^22^ At the same time, he acknowledged that Chinese medicine's insufficient scientific backing could cause errors in diagnosis and treatment, which served as an objective reason for the scepticism and distrust among biomedical doctors. For these reasons, Boldyrev expressed strong support for Mao's directive issued in June 1954, which called for establishing institutes dedicated to studying and researching Chinese medicine. These institutes would aim to uncover the essence of Chinese medicine and define its theoretical foundations.^23^ Consequently, the Chinese Medicine Research Institute under the Ministry of Health was established on December 19, 1955.

Boldyrev argued that it was the patriotic responsibility of both Chinese and Western medical professionals in China to unify modern biomedicine with traditional Chinese medicine, thereby creating a unified national medical science of their own. This new system would integrate the most advanced knowledge and achievements of modern medicine alongside all the best aspects of Chinese medicine. He identified acupuncture as an ideal starting point for this integration, stating:

It can be confidently asserted that an in-depth study of acupuncture, which holds high esteem in China, could open a completely new chapter in modern medical theory. This [new knowledge] would require a reassessment of existing theories on the neural regulation of bodily functions in both healthy individuals and patients.^24^

**Figure 3.**
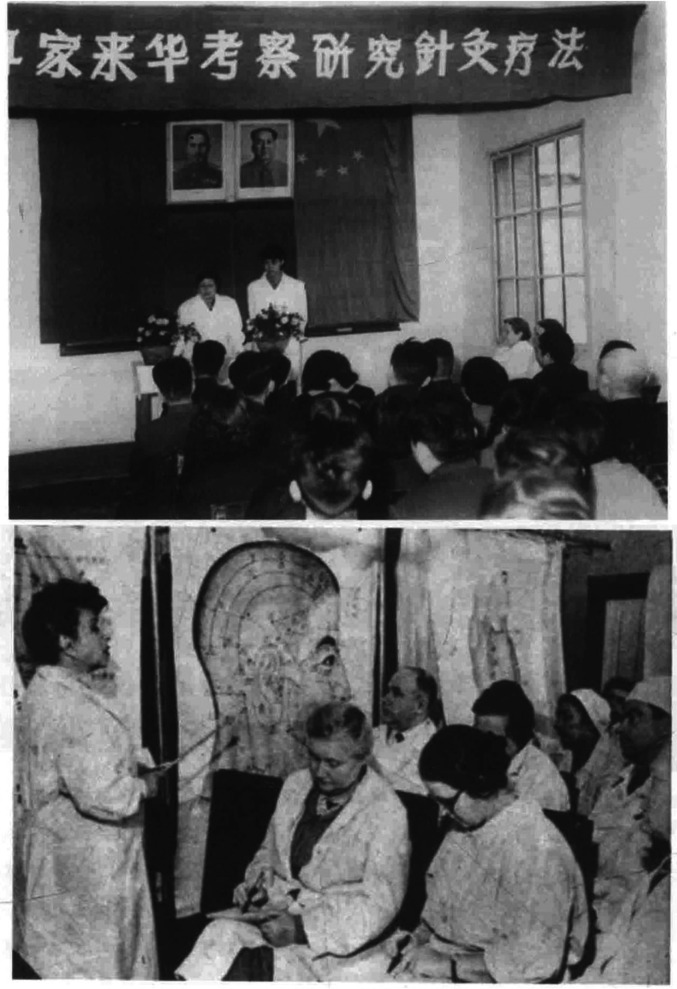
Soviet experts studying acupuncture (quoted from *Chinese Medicine Journal*, March 1957).

As the leader of the Soviet expert group, Boldyrev organised all Soviet medical professionals in Beijing to study and research acupuncture.^25^ In 1956, under the Sino-Soviet Scientific and Technological Cooperation Agreement, the USSR sent three neurologists to the Chinese Medicine Research Institute for a three-month investigation and study of acupuncture (Figure 3).^26^

Acupuncture had been utilised by the CCP's military since 1945. After 1949, under the widespread influence of Sovietisation, Soviet Pavlovian neuropathology emerged as the dominant theoretical framework in Chinese medical science.^27^ Recognised as a component of national cultural heritage that could be interpreted through the scientific lens of Pavlovian neuropathology and a treatment modality that was highly praised and studied by Soviet experts, acupuncture gained strong support from the Chinese state. In April 1958, Mao inquired about the progress made by Soviet experts in learning acupuncture and their efforts to promote it upon returning to the USSR. After hearing the report, Mao praised acupuncture, declaring, ‘Acupuncture has great value!’^28^ From this series of events, the reshaped basic theories of Chinese medicine came to be dominated by acupuncturists and were built upon the principles of acu-tracts theory and acupuncture-based therapeutics.

The vision of medical integration, however, was not monolithic. While both Boldyrev and Mao supported the integration of Chinese medicine with biomedicine, their conceptions of how exactly such integration would be best achieved differed in the details. Boldyrev emphasised that Chinese medicine practitioners should study modern biomedicine, including learning general biological knowledge, laboratory testing techniques, and clinical examination methods. He also criticised the traditional apprenticeship model, where newer practitioners would study under experienced grassroots practitioners, as an unreasonable method for training future doctors. A central reason for his objection was his belief that such apprenticeship structures failed to ensure the proper ideological education, which he considered indispensable.^29^ However, two months after Boldyrev's suggestions, in November 1954, Mao initiated the ‘Western medicine doctors should study Chinese medicine’ movement, during which Chinese medicine practitioners served as teachers for biomedical doctors.^30^ By 1956, Chinese medicine practitioners were also allowed to mentor apprentices, signifying a methodological shift in how the integration of Chinese and Western medicine was to be undertaken. This shift marked China's redirection of its medical approach and distanced it from Soviet methodologies.

## Suggestions on epidemic prevention

**Figure 4.**
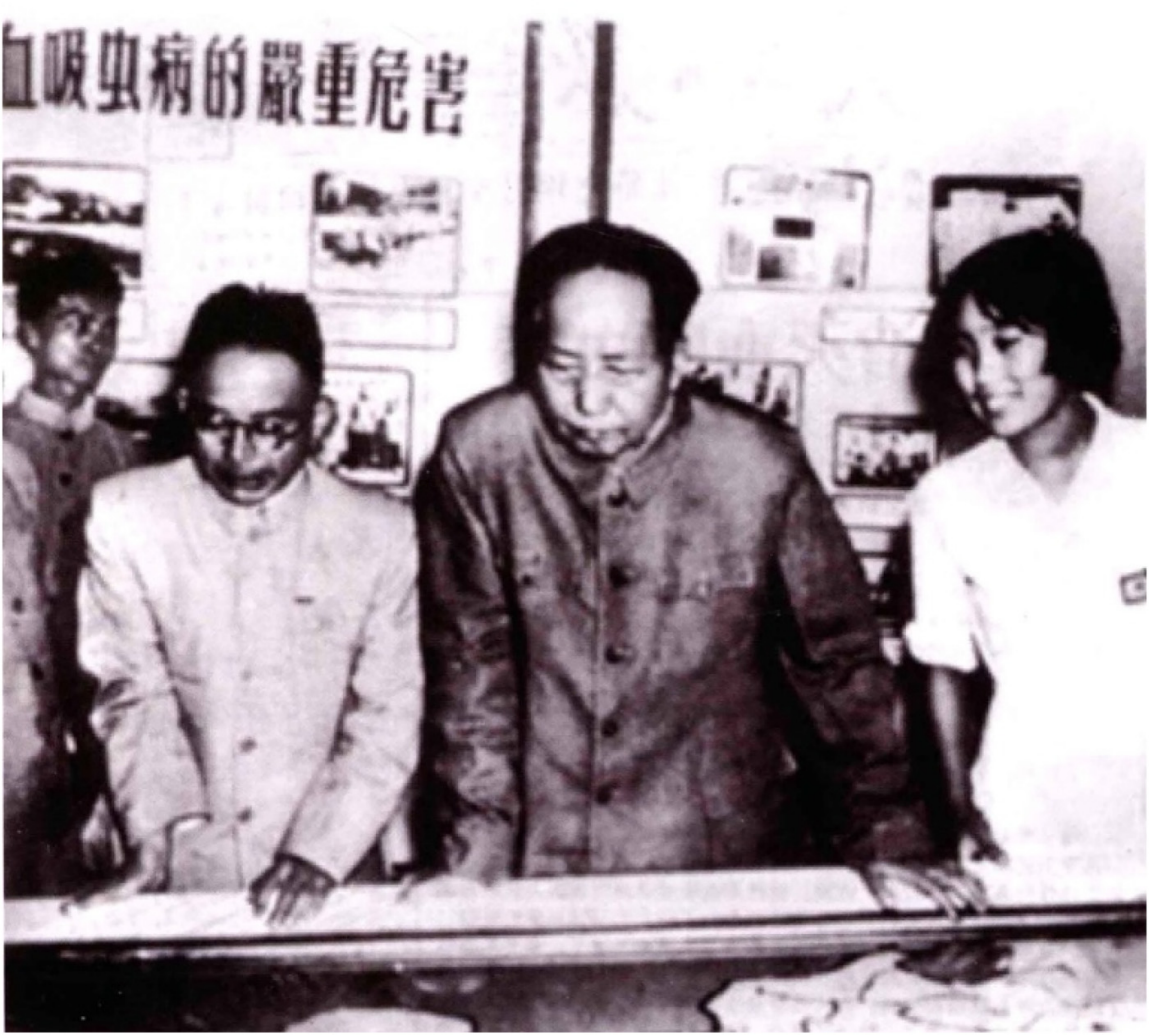
Mao Zedong inspecting schistosomiasis prevention and control work in Anhui Province, 1958.

Under Soviet influence, the PRC's first National Health Conference, held in August 1950, adopted ‘Prevention First’, ‘Unite Chinese and Western Medicine’, and ‘Serve the Workers, Peasants, and Soldiers’ as the three key principles of health work. These principles reflected the USSR's longstanding prioritization of epidemic prevention, which had been central to Bolshevik health policy since 1919. Following China's entry into the Korean War, in February 1952, the PRC and the Democratic People's Republic of Korea accused the United States of conducting bacterial warfare by spreading biological toxins and infectious agents through northern Korea and China. In response to these biological threats, the PRC launched a nationwide ‘Patriotic Hygiene Campaign’, mobilising the population in a massive public health initiative.^31^ Among the infectious diseases targeted in this campaign were those referred to as the ‘Gods of Plague’, including schistosomiasis, plague, malaria, and encephalitis (Figure 4).

In light of the growing concern for epidemic prevention, Boldyrev emerged as a key advisor, yet his role has been largely overlooked in previous research on epidemic control during 1950s China.^32^ Boldyrev was a seasoned expert in combating epidemics such as plague, malaria, and encephalitis, which were also prevalent in the USSR. The USSR's effective plague prevention measures, implemented in China since 1947, had proven largely successful, with only 31 cases of plague reported across China by 1955. Despite this progress, fears of biological warfare remained a contemporary bogeyman. Although modern scholars generally concluded that the United States’ biological attacks were unfounded, in the 1950s, the PRC acted as though such threats were real. Within this context, Boldyrev fully believed in the potential dangers and proposed in January 1956, ‘As part of the overall national air defence plan, measures should be implemented to protect against the most aggressive capitalist countries deploying plague as a weapon to infect the population’.^33^

Although well-versed in many endemic infectious diseases, Boldyrev had limited knowledge of schistosomiasis since it was not present in the USSR. To bridge this knowledge gap, he relied on Chinese scientists to study the disease's theoretical aspects and conducted field research on it to gain practical insights. In the winter of 1955, Boldyrev spent 29 days investigating the Yangtze River basin, a region heavily affected by schistosomiasis, submitting a 20,000-word report detailing strategies for its eradication thereafter.^34^ In his report, Boldyrev expressed confidence that, ‘under the direct leadership of the CCP Central Committee’, ‘without a doubt, the Chinese people would eradicate schistosomiasis, just as they have eliminated many other enemies’.^35^ He emphasised that the recent completion of agricultural collectivisation and the growth of agricultural cooperatives provided a solid foundation for mobilising the rural population. He anticipated that ‘with each successful harvest, farmers within the cooperatives would become stronger and more capable of implementing widespread efforts to prevent and control schistosomiasis, without relying on special financial assistance from the state’.^36^

Beyond helping Boldyrev better understand schistosomiasis itself, his time doing fieldwork in China also reshaped his views on how to treat the disease more universally. During his visit to the Yangtze delta, Boldyrev emphasised the limitations of antimony-based drugs commonly used at the time and advocated for a greater focus on Chinese herbal remedies. He criticised the prevailing research focus on identifying herbs that could directly kill schistosomes, arguing that this approach overlooked the possibility that herbs might work indirectly by addressing the toxic byproducts of schistosome activity. Boldyrev suggested that researchers study Chinese medicine within the context of the methods employed by its practitioners, as these methods may reveal the key to a treatment's overall effectiveness. He believed that the most effective treatment strategy would combine antimony-based drugs with detoxifying Chinese herbal remedies. He also praised the eradication methods developed by the Wuxi Schistosomiasis Prevention and Control Institute, such as ones employing tea seed cakes,^37^ which are poisonous to disease-carrying snails, as fertiliser in paddy fields. Boldyrev also noted an innovative practice in Nanjing and Hangzhou where human faeces were stored in covered containers and mixed with urine at a 1:3–1:5 ratio to create a mixture that would kill schistosome eggs.^38^

Eventually expanding his focus beyond combating schistosomiasis directly, Boldyrev became a strong proponent of the Chinese government's mass campaign to swiftly eradicate the ‘four pests’ of the country, namely rats, flies, mosquitoes, and sparrows. He emphasised that this eradication campaign of transmission vectors would be essential to the successful eradication of not only schistosomiasis, but also encephalitis, malaria, and plague. Boldyrev introduced a Soviet chemical method for mosquito control that involved positioning a large barrel of petroleum above infested water. A small hole pierced in the bottom of the barrel allowed a slow, steady drip of petroleum to spread across the surface, forming a thin film that suffocated mosquito larvae. In areas where targeted eradication was unfeasible, Boldyrev suggested using aeroplanes to mist the land with pesticide. He assured Chinese officials, ‘If aeroplanes are not currently available, 15 planes can be requested from the USSR’.^39^

## Conclusion

During China's critical nation-building of the 1950s, Tikhon Efimovich Boldyrev contributed to the country's health policy in ways that highlighted the Sino-Soviet collaboration. Even after Boldyrev's departure from China in 1956, he persisted in his efforts to enhance the global recognition of China's public health advancements. In 1957, as the USSR's representative to the International Quarantine Committee of the World Health Organisation (WHO), he introduced China's comprehensive schistosomiasis control measures at the 10th WHO Conference in Geneva.^40^ His presentation drew considerable attention, sparking international interest in the PRC's innovative disease prevention strategies. Boldyrev passed away in 1984 and was laid to rest in the columbarium of Moscow's Vagankovo Cemetery.
